# Multi-level barriers to fruit and vegetable waste awareness and management among retailers in the City of Johannesburg Metropolitan Municipality Region F: a Social Ecological Model lens

**DOI:** 10.3389/fpubh.2026.1872901

**Published:** 2026-07-06

**Authors:** Mpinane Flory Senekane, Cavin Omphemetse Moreetsi

**Affiliations:** Department of Environmental Health, Faculty of Health Sciences, University of Johannesburg, Johannesburg, South Africa

**Keywords:** awareness, formal and informal retailers, fruit and vegetable waste, practices, Social Ecological Model

## Abstract

**Introduction:**

Fruit and vegetable waste (FVW) poses environmental, economic, and public health challenges, particularly in urban African contexts where formal and informal retail coexist. This study aimed to determine and describe FVW awareness and management among retailers in the City of Johannesburg Metropolitan Municipality Region F, and to interpret the findings through the Social Ecological Model (SEM).

**Methods:**

A quantitative survey was conducted with 118 informal and 43 formal retailers, collecting data on socio-demographics, awareness, practices, and influencing factors. Chi-square tests examined associations between demographics and awareness.

**Results:**

Informal retailers had basic FVW knowledge but poor recognition of FVW as a global concern and a strong disposal-only mindset. Formal retailers showed higher awareness yet shared the disposal misconception. Infrastructure access was absent for informal traders, and government support was low for both sectors. Chi-square tests showed that age, gender, and education significantly influenced informal retailers’ awareness, whereas no socio-demographic factor affected formal retailers’ awareness.

**Discussion:**

Applying the SEM demonstrated compounded multi-level barriers for informal retailers and organizational strengths with persistent gaps for formal traders. Achieving effective FVW management requires integrated interventions across intrapersonal, interpersonal, organizational, community, and policy levels, moving beyond a disposal-centric approach.

## 1 Introduction

Food waste is one of the most critical sustainability issues experienced in the twenty-first century, with approximately one-third of all food intended for human consumption lost or wasted annually (1, 2). Within this context, fruits and vegetables (FV) are among the most wasted food types, accounting for a large portion, with estimates disclosing that nearly 44% of all FV produced in South Africa are wasted (3). The magnitude of this waste underscores the need for increased research on fruit and vegetable waste (FVW).

FVW disposed of in landfills undergo anaerobic decomposition, releasing greenhouse gases like methane (CH₄) and other harmful emissions such as ammonia and volatile organic compounds (VOCs). These contribute to climate change and air pollution (4). Landfill leachate and gases produced by the decomposition of organic waste can contaminate soil and water resources. This leads to ecosystem degradation and increases the risk of waterborne pollutants entering surface and groundwater (5). Additionally, FVW creates conditions that promote the presence of vectors, such as flies and rodents, which can increase the risk of pathogen transmission and vector-borne diseases in neighboring communities (4).

FVW remains a persistent challenge, particularly in sub-Saharan African cities, due to the coexistence of formal and informal retail systems. The informal FV retail sector plays a critical role in urban food distribution, ensuring that low-income communities have access to affordable, fresh produce. However, this sector is often characterized by poor infrastructure, low barriers to entry, and marginalization (6–8). These factors have been associated with the significant levels of FVW observed within the sector.

In contrast to the informal FV retail sector, the formal FV retail sector generally operates within structured supply chains, supported by established infrastructure, regulatory oversight, and greater access to financial and institutional resources. However, the challenge of FVW experienced in this sector is mainly due to Strict FV grading standards, demand forecasting errors, and supply chain inefficiencies. These challenges are further exacerbated by prevailing economic conditions in Sub-Saharan African countries, where consumers tend to favor more affordable FV sold in informal markets.

The City of Johannesburg Metropolitan Municipality (COJMM) Region F (one of South Africa’s economic nuclei) is a microcosm of this dual retail landscape, where formal and informal FV retailers coexist. The COJMM Region F is characterized by a dense population, cultural diversity, and significant socio-economic inequality (9). Understanding how retailers in the COJMM Region F perceive and manage FVW will aid in developing effective targeted interventions. Existing literature has primarily examined the formal and informal FV sectors separately, with few studies applying comprehensive theoretical frameworks to understand the complex factors that influence FVW management behavior across these sectors.

In addressing this gap, this study applies the Social Ecological Model (SEM) as an interpretive framework to understand FVW awareness and management among formal and informal FV retailers in the COJMM Region F. SEM posits that individual behaviors are influenced by multiple, interacting levels which are, intrapersonal (knowledge, attitudes, skills), interpersonal (social networks, relationships), organisational (policies, practices, structures), community (infrastructure, cultural norms, local context), and public policy (laws, regulations, government support). SEM highlights that behavior is influenced not only by personal factors but also by broader social, organizational, and policy environments in which individuals operate. The model’s strength lies in its recognition that effective behavioral change requires interventions across multiple levels concurrently, rather than focusing on individual awareness (10, 11).

The application of SEM to FVW management provides a more nuanced understanding of why retailers manage FVW effectively or fail to do so. For example, an informal retailer may know that proper waste disposal is important (intrapersonal) but lacks access to municipal bins (community) and has no knowledge of relevant municipal waste management by-laws (policy). A formal retailer may have a FVW policy (organizational) yet fail to engage with local FVW reduction initiatives (community). Through examining how factors at different levels interact, the SEM provides a framework for designing holistic, context-appropriate, and effective interventions.

This study, therefore, aims to determine and describe the awareness and management of FVW by formal and informal FV retailers in the COJMM Region F by assessing the level of awareness of FVW, describing current FVW management practices employed by these retailers, and identifying key factors influencing FVW generation. Lastly, these findings will then be interpreted through the lens of the SEM to produce multi-level recommendations.

## 2 Materials and methods

### 2.1 Description of study area

**Figure 1:**
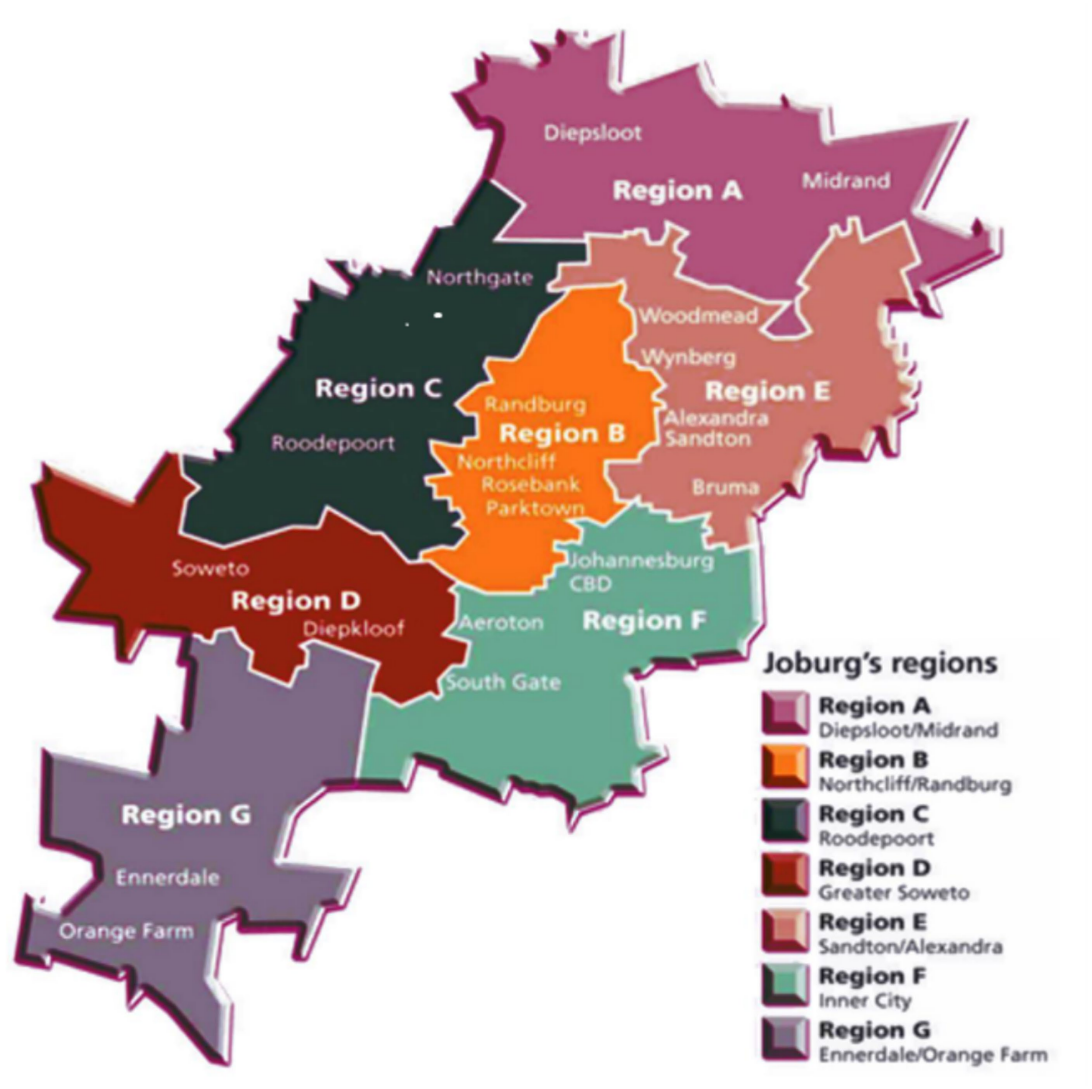
City of Johannesburg metropolitan municipality (33).

The study was carried out in Region F of the City of Johannesburg Metropolitan Municipality (COJMM), located in Gauteng Province (see Figure 1). This region encompasses the inner city of Johannesburg and functions as the historical and economic hub of the metropolitan area. This area was purposively selected for its clear administrative boundaries, coexistence of formal and informal retail, and documented waste management challenges. It has an estimated population of approximately 658,000 residents, representing about 13.3% of the city’s total population. The demographic profile is largely youthful and economically active, with 44% of residents aged 25–44. From an economic perspective, Region F is the leading contributor to Johannesburg’s gross domestic product and accounts for a substantial share of employment within the metro. Additionally, it is estimated that around 10,000 street traders operate within the inner city, collectively generating roughly R4.2 billion per year (9).

### 2.2 Study design

A quantitative descriptive design, using a non-experimental survey, was employed to determine and describe awareness and management of FVW among formal and informal FV retailers.

### 2.3 Study population

This study’s population includes both formal franchise supermarkets (formal retailers) and informal fruit and vegetable (FV) retailers. The targeted population consisted of 61 formal outlets and approximately 1,000 informal retailers. Specifically, Region F had 61 formal franchise supermarkets. The precise number of informal retailers who solely sell FV remains uncertain due to ongoing fluctuations. However, an expert familiar with these retailers estimated that there are around 1,000 informal FV sellers.

### 2.4 Sampling

Purposive sampling was employed to select retailers involved in daily FV sales and handling in Region F of the COJMM. This method was chosen because the target population is highly diverse in operation and poorly documented, particularly within the informal sector, where most retailers are unregistered, mobile, and not listed in municipal business databases. Consequently, probability sampling was not feasible. The researchers deliberately concentrated on two groups with significant exposure to FVW generation: informal fruit and vegetable retailers operating in public trading areas and formal franchise supermarkets in the region. This strategy ensured the inclusion of participants with substantial experience in FVW management, thereby improving the relevance and depth of the results.

### 2.5 Sample size estimation

Sample size determination was conducted using Epi Info version 7, based on a 90% confidence level and a margin of error of ±7%. The selected confidence level reflected a balance between statistical reliability and the practical challenges associated with field data collection, particularly when working with informal retailers who are often mobile and not easily accessible during business hours. The calculated sample sizes were 121 for informal retailers and 43 for formal retailers. The final achieved sample included 118 informal retailers and all 43 formal retailers.

### 2.6 Pilot study

A pilot study was conducted in Region F of the COJMM prior to the main data collection phase. The pilot study served to refine the questionnaire by assessing the clarity, relevance, and comprehensiveness of the items. The instrument was based on the Social Ecological Model and relevant literature on FVW. Given that the questionnaire comprised items measuring multiple distinct constructs across different levels of the SEM, internal consistency reliability was not considered appropriate for the instrument as a whole. The pilot sample comprised 10 informal FV retailers and 5 formal FV retailers, all of whom completed the same questionnaire intended for use in the main study, according to their respective categories.

Ethical protocols applied in the main study were consistently followed during the pilot. Participants were informed about the purpose of the research, provided with an information sheet, and required to give informed consent before participation. The pilot process was instrumental in identifying ambiguous or poorly structured items, allowing for necessary revisions to improve the clarity and comprehensiveness of the questionnaire. To prevent potential bias, individuals who participated in the pilot were excluded from the main study.

### 2.7 Data collection

Data was collected using a structured questionnaire administered by the researchers to both formal and informal FV retailers. The instrument was structured into two primary sections: Section A gathered socio-demographic and operational data (gender, age, and education level). Section B entailed questions about awareness and management of FVW.

The questionnaire was structured primarily using a Likert-scale format. This approach was selected due to the fast-paced nature of retail environments, where participants are often occupied with customer interactions. The use of the Likert-scale format enabled efficient data collection, as such questions are straightforward, require minimal time to complete, and are well-suited to respondents with limited availability. In addition, other questions were presented in multiple-choice and dichotomous formats.

### 2.8 Data analysis

The questionnaires’ data were analyzed using IBM SPSS Statistics software, version 30.0. Data from Google Forms was converted into an Excel sheet and then imported into SPSS. The initial step involved cleaning the data by removing missing values, typographical errors, duplicates, and anomalies, which is essential for ensuring data quality and avoiding unreliable results (12). Descriptive analysis was conducted to present the findings with percentages.

#### 2.8.1 Hypothesis development for chi-square tests

To determine whether demographic characteristics influence awareness of FVW, Pearson’s chi-square tests of independence were conducted. The independent variables were age group, gender, and educational level. The dependent variables were the five intrapersonal awareness questions from section B of the questionnaire. The assumptions of independence and categorical measurement were met. Where sparse cell counts occurred, categories were reviewed to ensure the analysis was appropriate.

The following hypotheses were tested separately for informal and formal retailers, and statistical significance was set at *p* < 0.05 (see Table 1).

**Table 1:** Chi-square test parameters.

Awareness variable	Demographic variable	Hypothesis tested	Purpose
Knowledge of what FVW waste is	Age groups	H01a/HA1a	Tests whether awareness levels are influenced by age.
Awareness of the impact of poor FVW management			
FVW is a global concern			
Minimizing FVW is good practice	Gender	HO1b/HA1b	Tests whether awareness levels are influenced by gender.
Disposal FVW is the only effective waste management method.	Educational level	HO1c/HA1c	Tests whether awareness levels are influenced by educational level

*H_0_:* There is no significant difference between retailers' demographic characteristics (age, gender, and educational level) and FVW awareness.

*H_1_:* There is a significant association between retailers' demographic characteristics (age, gender, and educational level) and FVW awareness.

### 2.9 Application of the Social Ecological Model

The Social Ecological Model (SEM) was employed as the theoretical framework to interpret the multi-level factors influencing FVW awareness and management among formal and informal retailers. This mapping enabled systematic analysis of how factors at different levels interact to shape FVW-related outcomes for both retailer groups. To apply the SEM to this study, questions were mapped to each level.

However, some sets of questions were not similar across the two retailer groups because the groups operate under fundamentally different structural conditions. Informal retailers are sole proprietors with no formal policies, training programs, or induction processes; consequently, the organizational level is effectively absent for them. In contrast, formal retailers work within established corporate structures that include active food waste policies, employee training, and standard operating procedures. For example, interpersonal influences were measured using the same questions, but community and policy questions were adapted to reflect the distinct realities of each group.

### 2.10 Ethical clearance

The study received approval from the University of Johannesburg’s Research Ethics Committees (REC-3201-2024). Permission was also granted by the City of Johannesburg Metropolitan Municipality’s Department of Corporate & Shared Services in the office of the Group Head: Group Human Capital Management, as well as by the Johannesburg Property Company (JPC). The study was carried out in accordance with local laws and institutional policies. Before starting, participants were informed of the study’s purpose, nature, and scope, as well as how data would be collected, through a study information letter. Throughout the process, participants received all necessary information about the study. They were also briefed on the researcher’s role. Participants were asked to sign a consent form authorizing the researcher to include them in the study.

## 3 Results

The results section comprises two sets of data, one for informal retailers and another for formal retailers. Phase 1 presents the results for informal retailers, and Phase 2 presents the results for formal retailers.

### 3.1 Phase 1: informal retailers

#### 3.1.1 Socio-demographic characteristics of informal retailers

A total of 118 informal retailers were included in the study sample. As indicated in Table 2, the majority of participants were male (77; 65.3%), while 41 respondents were female (34.7%). In terms of age distribution, most participants were between 30 and 49 years (67.8%), whereas only a very small proportion fell within the 70–89 age category (0.8%). With respect to educational attainment, the largest share of respondents had completed primary education (39%), while only a limited number had obtained tertiary qualifications (3.4%).

**Table 2:** Socio-demographic characteristics of informal retailers.

Socio-demographic factors		Frequency (*n*)	Percentage (%)
Gender	Male	77	65.3
	Female	41	34.7
Age	18–29	24	20.3
	30–49	80	67.8
	50–69	13	11.1
	70–89	1	0.8
Highest educational level	Never went to school	28	23.7
	Primary	46	39.0
	Secondary	40	33.9
	Tertiary	4	3.4

#### 3.1.2 Informal retailers SEM findings

A Intrapersonal level (awareness)

Five intrapersonal questions measured knowledge, attitudes, and beliefs about FVW.

Participants demonstrated high familiarity with FVW: 78.8% agreed and 20.3% strongly agreed, while 0.9% remained neutral and none expressed disagreement. Awareness of the impacts of poor FVW management was more varied, with 39.8% agreeing and 33.1% disagreeing, and very few (1.7%) strongly agreeing. When asked whether FVW is a global concern, responses leaned toward disagreement (31.4% disagree; 22% strongly disagree), although 28.8% agreed.

Most participants (79.7%) viewed minimizing FVW as good practice, with 17.8% neutral and negligible disagreement. However, a large majority (83.9%) believed that disposal is the only effective waste management method, while 11% were neutral, and none strongly disagreed (see Table 3).

**Table 3:** Intrapersonal level findings (informal retailers).

Question	Agree (%)	Strongly agree (%)	Disagree (%)	Strongly disagree (%)	Neutral (%)
Knowledge of what FVW is	78.8	20.3	0	0	0.9
Awareness of the impact of poor FVW management.	39.8	1.7	33.1	6.8	18.6
FVW is a global concern	28.8	0	31.4	22.0	17.8
Minimizing FVW is good practice	79.7	1.7	0.8	0	17.8
Disposal FVW is the only effective waste management method.	83.9	3.4	1.7	0	11.0

B Interpersonal level

One question assessed compliance with advice from government officials (see Table 4).

**Table 4:** Interpersonal level findings (informal retailers).

Question	Agree (%)	Strongly agree (%)	Disagree (%)	Strongly disagree (%)	Neutral (%)
Comply with advice from government officials	79.7	13.6	1.6	0	5.1

Participants were asked whether they complied with advice from government officials on managing FVW. The majority (79.7%) agreed, while only 1.7% disagreed, and no one strongly disagreed (0%).

C Community level

Four questions captured infrastructure access, market forces, and physical practices (see Table 5).

**Table 5:** Community level findings (informal retailers).

Question	Agree (%)	Strongly agree (%)	Disagree (%)	Strongly disagree (%)	Neutral (%)	Yes/no (%)
Access to waste management infrastructure (e.g., bins)						Yes = 4.3
						No = 95.7
Customer demand fluctuation affects FVW generation	40.1	58.7	1.2	0	0	
Store fruit and vegetables in suitable conditions	32.2	10.2	44.9	0	12.7	
FVW must mix with other waste types	42.4	1.7	35.6	0	20.3	

Most participants (95.7%) reported lacking access to waste management infrastructure such as composting bins, with only 4.3% indicating availability. Customer demand fluctuations were widely perceived to influence FVW generation, with 58.7% strongly agreeing and 40.1% agreeing. In terms of storage practices, a larger proportion (44.9%) indicated that FV were not kept under suitable conditions, compared to 32.2% who reported appropriate storage. Regarding disposal practices, 42.4% agreed that FVW should be mixed with other waste streams, while 35.6% disagreed.

D Public policy level

Five questions addressed by-law awareness, government support, and information access (see Table 6).

**Table 6:** Public policy level findings (informal retailers).

Question	Agree (%)	Strongly agree (%)	Disagree (%)	Strongly disagree (%)	Neutral (%)	Yes/no OR multiple choice (%)
Biggest obstacle to managing FVW						Financial resources = 69.5
						Lack of government support = 16.9
						cutting down on ordering = 11.3
						No obstacles = 2.3
Governmental support for FVW management						Yes = 12.7
						No = 87.3
Informed of ways to minimise FVW	0	1.7	81.4	3.4	13.5	
Well-informed on proper FVW disposal	28.8	1.7	44.9	0	24.6	
Awareness of COJMM waste management by-laws	11	1.7	54.2	0	33.1	

Participants identified financial constraints as the primary barrier to effective FVW management (69.5%), followed by limited government support (16.9%). The vast majority (87.3%) reported not receiving any form of governmental assistance. Awareness of strategies to reduce FVW was generally low: 81.4% reported a lack of knowledge, 13.5% remained neutral, and only 1.7% strongly agreed they were informed. Similarly, knowledge of proper disposal practices was limited: 44.9% disagreed that they were well-informed, compared with 28.8% who agreed. Awareness of the City of Johannesburg Metropolitan Municipality’s waste management by-laws was also low, with 54.2% indicating disagreement and 33.1% expressing neutrality.

#### 3.1.3 Chi-square test results for intrapersonal awareness questions (informal retailers)

Pearson’s chi-square tests were conducted to examine associations between demographic variables (age, gender, and education) and the five intrapersonal awareness questions (see Table 7).

**Table 7:** Chi-square results for informal retailers.

Intrapersonal question	Age *p*-value	Gender *p*-value	Education *p*-value
Knowledge of what FVW is	0.042*	0.750	0.059
Awareness of the impact of poor FVW management	<0.001*	<0.001*	<0.001*
FVW is a global concern	<0.001*	0.001*	<0.001*
Minimizing FVW is good practice	0.001*	0.140	<0.001*
Disposal FVW is the only effective waste management method	0.802	0.005*	0.092

^*^p < 0.05—statistically significant.

Chi-square test results were analyzed across age, gender, and educational level. For age, four out of five variables showed statistically significant differences in awareness, leading to the rejection of the null hypothesis. In terms of gender, three out of five variables indicated significant differences between male and female informal retailers, also resulting in rejection of the null hypothesis. Similarly, educational level showed significant variation in three out of five variables, confirming that awareness differs by educational background.

Overall, these findings indicate that age, gender, and education significantly influence FVW awareness among informal retailers.

### 3.2 Phase 2: formal retailers

#### 3.2.1 Socio-demographic characteristics of formal retailers

As indicated in Table 3, 43 formal retailers participated in the study, comprising 25 females (58.1%) and 18 males (41.9%). The majority of respondents were in the 18–29 age group (60.5%), while the least represented group was 50–65 years, with only 1 participant (2.3%). Regarding educational attainment, most participants had completed secondary education (60.5%), with no respondents reporting either no formal schooling or only primary-level education. A substantial proportion (39.5%) had achieved tertiary-level qualifications (see Table 8).

**Table 8:** Socio-demographic characteristics of formal retailers.

Socio-demographic factors		Frequency (*n*)	Percentage (%)
Gender	Male	18	41.9
	Female	25	58.1
Age	18–29	26	60.5
	30–49	16	37.2
	50–65	1	2.3
Highest educational level	Never went to school	0	0
	Primary	0	0
	Secondary	26	60.5
	Tertiary	17	39.5

#### 3.2.2 Formal retailers SEM findings

E Intrapersonal level (awareness)

Five intrapersonal questions measured knowledge, attitudes, and beliefs about FVW (see Table 9).

**Table 9:** Intrapersonal level findings (formal retailers).

Question	Agree (%)	Strongly agree (%)	Disagree (%)	Strongly disagree (%)	Neutral (%)
Knowledge of what FVW is	9.3	90.7	0	0	0
Awareness of the impact of poor FVW management.	37.2	46.5	0	0	16.3
FVW is a global concern	23.3	16.2	25.6	20.9	14.0
Minimizing FVW is good practice	55.8	39.5	0	0	4.7
Disposal FVW is the only effective waste management method	65.1	32.6	0	0	2.3

Most formal retailers demonstrated high awareness of FVW, with 90.7% strongly agreeing and 9.3% agreeing that they know what FVW is. Awareness of the impacts of poor FVW management was also high, with 46.5% strongly agreeing and 37.2% agreeing. Perceptions of FVW as a global issue were more divided, with the largest proportion disagreeing (25.6%), followed by 23.3% who agreed and 20.9% who strongly disagreed.

In contrast, most participants viewed minimizing FVW positively, with 55.8% agreeing and 39.5% strongly agreeing, and no disagreement was recorded. However, a strong majority (65.1% agreed; 32.6% strongly agreed) also believed that disposal is the only effective method of managing FVW.

F Interpersonal level

One question assessed compliance with advice from government officials (see Table 10).

**Table 10:** Interpersonal level findings (formal retailers).

Question	Agree (%)	Strongly agree (%)	Disagree (%)	Strongly disagree (%)	Neutral (%)
Comply with advice from government officials	53.5	39.5	0	0	7.0

High compliance with advice from government officials was reported, with most respondents agreeing (53.5%) and strongly agreeing (39.5%).

G Organizational level

Six questions captured policies, training, and store practices (see Table 11).

**Table 11:** Organizational level findings (formal retailers).

Question	Agree (%)	Strongly agree (%)	Disagree (%)	Strongly disagree (%)	Neutral (%)	Yes/no OR Multiple Choice (%)
The store has an active food waste policy	39.5	58.10	0	0	2.4	
Workers trained on policy implementation	62.8	25.6	2.0	0	9.6	
Inducted on ways to minimize FVW	69.8	27.9	2.3	0	0	
FVW must mix with other waste types	0	4.7	9.2	14.0	72.1	
Management of FVW						Dispose in a Disposal Bin = 94.0
						Distribute to Processing Plants = 4.7
						Donate = 1.3
Unpurchased FVW stock management						Dispose = 18.6
						Donate = 23.3
						Mark down = 37.2
						Return to storage = 11.5
						Rework = 9.4

Most formal retailers indicated that their stores have an active food waste policy, with 58.1% strongly agreeing and 39.5% agreeing. Similarly, the majority reported that employees are trained on policy implementation (62.8% agree; 25.6% strongly agree). Induction on FVW minimization practices was also widely reported, with 69.8% agreeing and 27.9% strongly agreeing. When asked whether FVW should be mixed with other waste types, most respondents were unsure, as indicated by their selection of the neutral option (72.1%). Interestingly, a high proportion of respondents disagreed (9.2%) and strongly disagreed (14%).

In terms of waste handling practices, most retailers disposed of FVW via waste bins (94%), while a small proportion sent waste to processing facilities (4.7%) or donated it (1.3%). However, management of unsold stock varied, with markdowns being the most common strategy (37.2%), followed by donation (23.3%). Smaller proportions reported disposal (18.6%), returning stock to storage (11.5%), or reworking products (9.4%).

H Community level

One question was used to assess the community level for formal retailers (see Table 12).

**Table 12:** Community level findings (formal retailers).

Question	Yes	No (%)
Local initiatives for FVW management	Yes = 51	No = 49

Although most formal retailers reported involvement in initiatives (51%), there is a need for greater participation, as many respondents did not participate (49%).

I Public policy level

Four questions assessed by-law awareness, government support, and information (see Table 13).

**Table 13:** Public policy level findings (formal retailers).

Question	Agree (%)	Strongly agree (%)	Disagree (%)	Strongly disagree (%)	Neutral (%)	Yes/no (%)
Awareness of COJMM waste management	27.5	6.9	11.6	0	54.0	
Well-informed on proper FVW disposal	58.1	34.9	7.0	0	0	
Governmental support received for FVW						Yes = 27.9
						No = 72.1
Need for improved government backup						Yes = 73,8
						No = 26.2

Over half of the participants (54%) reported uncertainty regarding their familiarity with COJMM by-laws, while 27.5% indicated awareness, and 11.6% expressed lack of awareness. In contrast, most participants reported being knowledgeable about proper FVW disposal methods, with 58.1% agreeing and 34.9% strongly agreeing.

A majority (72.1%) reported not receiving government support for FVW management, whereas 27.9% reported receiving some level of assistance. Accordingly, most participants (73.8%) expressed the need for improved government support, while a smaller proportion (26.2%) did not share this view.

#### 3.2.3 Chi-square test results for intrapersonal awareness questions (formal retailers)

Pearson’s chi-square tests were conducted to examine associations between demographic variables (age, gender, and education) and the five intrapersonal awareness questions (see Table 14).

**Table 14:** Chi-square results for formal retailers.

Intrapersonal question	Age *p*-value	Gender *p*-value	Education *p*-value
Knowledge of what FVW is	0.805	0.473	0.653
Awareness of the impact of poor FVW management.	0.158	0.155	0.504
FVW is a global concern	0.173	0.813	0.775
Minimizing FVW is good practice	<0.001^*^	0.073	0.876
Disposal FVW is the only effective waste management method	0.005^*^	0.805	0.480

^*^p < 0.05—statistically significant.

Regarding age, only 2 of 5 variables showed statistically significant differences in FVW awareness across age groups. However, no significant associations were observed for the remaining three awareness variables. Regarding gender and educational level, all *p*-values were above 0.05, leading to the acceptance of the null hypotheses and indicating no significant differences in awareness based on these variables. Therefore, these results show that age, gender, and the educational level of formal retailers do not affect the level of awareness they have regarding FVW.

## 4 Discussion

This study examined the awareness and management of FVW among formal and informal retailers in the COJMM Region F through the lens of the Social Ecological Model. The findings reveal distinct patterns across the two sectors: informal retailers face compounded barriers across multiple SEM levels, while formal retailers benefit from organizational supports but share a critical knowledge gap.

### 4.1 Informal retailers

#### 4.1.1 Intrapersonal level: knowledge, attitudes, and skills

The intrapersonal level captures individual knowledge, attitudes, and beliefs about FVW. Among informal retailers, basic awareness was high, as the majority reported that they knew what FVW constitutes. However, a deeper understanding revealed significant gaps. Only a minority reported being aware of the impacts of poor FVW management. Even more striking, only 28.8% recognized FVW as a global concern, while 53.4% disagreed or strongly disagreed. These findings align with those of Maphanga and Madonsela (13), who found that vendors demonstrated limited awareness of broader FVW environmental implications. A recent study similarly identified that informal retailers in Johannesburg displayed poor awareness of the global significance of FVW (14).

Critically, informal retailers agreed that disposal is the only effective waste management method, reflecting a profound knowledge gap regarding the waste hierarchy. This “disposal mindset” is consistent with findings across Sub-Saharan Africa, where informal vendors often default to discarding strategies due to a lack of awareness of alternative approaches such as composting, donation, or valorization (15). In contrast, positive attitudes toward waste minimization and proper disposal indicate that informal retailers are receptive to improved practices, provided enabling conditions are established. Similar findings were noted in a study in which informal traders stated their willingness to participate in recycling and composting programs, which should be carried out by market management in collaboration with local authorities (15).

Chi-square analysis indicated that age, gender, and educational level significantly influenced awareness among informal retailers. Age was associated with four of the five intrapersonal variables, including knowledge of FVW (*p* = 0.042), awareness of its impacts (*p* < 0.001), perception of FVW as a global issue (*p* < 0.001), and recognition of minimization as good practice (*p* = 0.001). Gender was significantly associated with awareness of impacts (*p* < 0.001), global concern (*p* = 0.001), and perceptions of disposal as the only management method (*p* = 0.005). Similarly, educational attainment was significantly linked to awareness of impacts, global concern, and minimization practices (all *p* < 0.001). These findings corroborate research that identified educational attainment as a key determinant of FVW awareness and management capacity among informal market actors (16).

#### 4.1.2 Interpersonal level: social networks and authority influence

The interpersonal level assesses how social interactions and advice-seeking shape behavior. Informal retailers reported complying with government officials’ advice concerning FVW management. This high compliance rate is noteworthy, particularly given that most respondents were unaware of the COJMM waste management by-laws and reported not being informed about ways to minimize FVW. This paradox suggests that informal retailers are willing to follow guidance when it is provided, but such guidance is rarely offered (15).

A study of informal fresh produce traders in Durban found that respondents primarily sought waste management information via radio (84.3%) and word of mouth (75.1%), with only 39.6% receiving it from municipal brochures (15). This suggests that municipal authorities in South Africa are underutilizing accessible communication channels. The high compliance rate observed in this study indicates that government officials could serve as effective messengers for waste management education, leveraging existing trust relationships, as research has identified “trusted messengers” as crucial for shaping behavior (17).

#### 4.1.3 Organizational level: absence of structured support

At the organizational level, there was a stark absence of informal retailers. As sole proprietors, they operate without formal policies, training programs, or quality control systems. The knowledge-practice gap reflected in their response, in which only a minority agreed that they store FVW suitably, underscores the absence of organizational support that would translate knowledge into consistent practice. This implementation deficit has been documented across Sub-Saharan Africa, where vendors possess adequate knowledge but cannot implement proper practices due to a lack of organizational structures and resources (18, 19). The finding that 81.4% disagreed that they were informed about ways to minimize FVW further underscores the complete absence of structured guidance.

#### 4.1.4 Community level: infrastructure, market forces, and physical environment

The community level revealed foundational barriers that transcend individual motivation. Access to waste management infrastructure was almost non-existent, as informal retailers reported lacking access to bins or similar facilities. A study of fresh produce markets in Dar es Salaam, Tanzania, similarly found “little formal provision for the collection, treatment and disposal of waste,” highlighting a systemic gap across East and Southern Africa (20). The absence of infrastructure may limit retailers’ ability to practice proper waste disposal, even where willingness exists, a structural barrier that no amount of individual education can overcome.

Customer demand fluctuation was identified as a major factor affecting FVW generation. This external market force complicates stock planning and may cause increased FVW generation, particularly for informal traders with limited capital and storage capacity. Research on Nigerian informal markets has identified demand variability as a key determinant of food-related waste (21).

The inefficient practice evident in storage practices reflects a lack of access to proper storage resources (cooling, shelving, pest protection) rather than an unwillingness. Regarding waste segregation, the general agreement that FVW should be mixed with other waste types when disposed of indicates a poor understanding of segregation principles. This aligns with the findings of Grangxabe et al. (19), who found that most informal retailers resort to informal waste disposal methods. The reported compliance with government official advice can influence this finding. COJMM informal retailers are more likely to adopt waste segregation practices when appropriate infrastructure (segregation bins) is provided, which informal retailers reported not having access to.

#### 4.1.5 Public policy level: by-law awareness and government support

The policy level revealed systematic failures. Awareness of COJMM waste management by-laws was extremely low. A study in Nquthu Municipality, KwaZulu-Natal, similarly found that small business owners frequently reported limited awareness and understanding of waste management laws, which hindered their ability to meet their legal obligations as waste generators (22). These findings suggest that policy communication challenges may extend beyond COJMM Region F.

Government support was perceived as minimal, as most informal retailers reported receiving no governmental assistance for FVW management, and identified financial resources as the biggest obstacle, followed by lack of government support. A minority of informal retailers reported being informed about ways to minimize FVW and being well-informed about proper disposal. These findings align with Dube et al. (23), who found that informal vendors face “multi-level exclusion” from education systems, social networks, organizational structures, community infrastructure, and policy frameworks.

### 4.2 Formal retailers

#### 4.2.1 Intrapersonal level: high awareness but shared misconceptions

Formal retailers demonstrated substantially higher awareness levels than their informal counterparts. Knowledge of FVW was near-universal, with general agreement. Awareness of the impacts of poor FVW management was also high. Positive attitudes toward minimization and proper disposal were similarly strong.

However, recognition of FVW as a global concern was mixed. This finding suggests that even well-resourced formal retailers may lack contextual awareness of the global scale of FVW issues. A study conducted in the United Kingdom found that formal retailers exhibited a homogenized awareness pattern but also possessed limited knowledge of the global significance of FVW (24).

Critically, formal retailers agreed or strongly agreed that disposal is the only effective waste management method, a finding almost identical to that of informal traders. This shared misconception is particularly concerning given the organizational resources available to formal retailers. A recent study examining the same study population characterized this as “fragmented governance across retail sectors, characterized by consistent dependence on disposal-centered practices and a lack of adoption of FVW valorization strategies” (25).

The chi-square analysis for formal retailers showed that only age was significant for two of five intrapersonal questions: minimization as good practice (*p* < 0.001) and disposal as the only method (*p* = 0.005). Gender and educational level showed no significant associations with any of the awareness questions (all *p* > 0.05). This homogeneity reflects the standardized training and corporate policies typical of formal retail environments, where individual demographic differences are subsumed by organizational norms.

#### 4.2.2 Interpersonal level: high compliance willingness

Formal retailers reported very high compliance with government officials’ advice. This finding is consistent with research conducted by Ghani et al. (26), which found that employee awareness training and compliance with regulatory guidance are among the most effective strategies to reduce food waste. The strong willingness to comply suggests that government-led initiatives would be well received by formal retailers, provided that such guidance is offered.

#### 4.2.3 Organizational level: policies and training, but disposal-dominant practices

The organizational level revealed both strengths and contradictions. Formal retailers demonstrated strong organizational support, as most reported that their stores have an active food waste policy, were inducted in ways to minimize FVW, and confirmed that workers are trained on policy implementation. However, despite these organizational advantages, actual waste-handling practices remained disposal-dominated. When dealing with FVW, the majority are disposed of, with only 4.7% of formal retailers distributing it to processing plants and 1.3% donating it. Management of unpurchased stock showed more variation: markdowns (37.2%), donation (23.3%), disposal (18.6%), and return to storage (11.5%). The finding that donation was more common than disposal for unpurchased stock is positive, but the continued reliance on disposal for FVW indicates that organizational policies are not translating into sustainable practices.

This finding may indicate that organizational policies are more strongly oriented toward compliance and documentation than systemic change. The finding that 72.1% of respondents were neutral or uncertain about whether FVW should be mixed with other waste types when disposed of indicates ongoing confusion about waste segregation, despite training programs.

#### 4.2.4 Community level: moderate local engagement

The community level showed moderate engagement: almost half of formal retailers reported no involvement in local initiatives for FVW management. This finding suggests missed opportunities for community-level collaboration, such as donating unsold produce to local food banks or participating in municipal composting schemes. This finding is also corroborated by the findings in the organizational level of formal retailers, where there is underutilization of donations and distribution to processing plants. Research on surplus retail food redistribution has demonstrated that structured partnerships between formal retailers and community organizations can achieve substantial sustainability gains (27). Similar models are emerging in South Africa, with initiatives like “Still Good” connecting consumers to discounted surplus groceries and FoodForward SA collecting nutritious surplus food from retailers for redistribution (28, 29).

#### 4.2.5 Public policy level: by-law uncertainty and government support gaps

The policy level revealed significant gaps. Awareness of COJMM bylaws was characterized by uncertainty. This high uncertainty rate is concerning, as formal businesses are expected to comply with municipal regulations. Government support was perceived as low for FVW management. Despite this, most formal retailers expressed the need for improved government backup. This finding, together with the willingness to comply with government advice, suggests that formal retailers would be receptive to increased government engagement. This finding also impedes targeted interventions to align retailer practices with circular-economy principles and the Sustainable Development Goals (SDGs). SDG 12 aims to ensure sustainable production and consumption patterns, and its third target (12.3) calls for halving per capita global food waste at the retail and consumer levels. African countries, including South Africa, have undertaken the “123 pledge,” which is a call for governments and any other agencies to prioritize reducing food waste (30).

Recent studies have shown that subjective norms and knowledge about FVW among retailers directly impact waste management. When retailers recognize that the irregular management of FVW in their daily operations has a significant negative impact and feel collectively responsible for it, they are motivated to act positively towards it, as they believe they can (31). This provides evidence that, through actions by authorities (such as educational programs and collaboration), retailers’ actions can serve as channels for positivity, contributing to the country’s achievement of its commitment under the 123-pledge. However, the gap between policy ambition and on-the-ground support for retailers remains substantial.

### 4.3 Socio-demographic influences: contrasting patterns across sectors

The chi-square analysis revealed fundamentally different patterns of demographic influence between sectors. For informal retailers, age, gender, and education significantly influenced awareness for most intrapersonal questions. This finding aligns with the broader literature on pro-environmental behavior, which consistently identifies education as a key determinant of environmental awareness (32). For formal retailers, by contrast, only age showed significance for two questions, while gender and education showed no significant associations.

This divergence reflects the standardized organizational environment of formal retail. In formal settings, employees receive uniform training, follow standard operating procedures, and operate under consistent policies, regardless of their individual demographic characteristics. In informal settings, where no such organizational standardization exists, individual characteristics exert a stronger influence on awareness and practices. This finding has important policy implications: interventions for informal retailers must be tailored to diverse demographic profiles, while formal retailers can be reached through uniform organizational channels.

### 4.4 Synthesis: compounded disadvantage and the need for multi-level interventions

The SEM reveals that informal traders face compounded disadvantages across all five levels: intrapersonal knowledge gaps are exacerbated by the absence of organizational support, inadequate community infrastructure, and poor policy dissemination. Formal retailers, while advantaged at the organizational level, share the critical misconception about disposal being the only effective method and face gaps at the community and policy levels. Effective FVW reduction requires interventions targeting multiple levels simultaneously. Individual education alone is insufficient without infrastructure, policy clarity, and organizational support. Similarly, organizational policies alone cannot succeed without addressing the intrapersonal knowledge gap about waste valorization. The findings underscore that moving “away from the bin” requires a coordinated, multi-level approach that recognizes the distinct realities of both formal and informal retail sectors.

## 5 Recommendations

The study’s recommendations are interconnected, but emphasis should be on interventions targeting the most urgent and fundamental barriers to FVW management. Key priorities include establishing accessible waste management infrastructure, enhancing communication of municipal waste bylaws, and providing targeted educational support for retailers. Tackling these barriers is essential because they serve as the foundation for successfully implementing other strategies like waste segregation, donation, and valorization.

### 5.1 Intrapersonal level

For informal retailers, the COJMM Environmental Health (EH) Department should develop targeted educational campaigns that explain not just what to do, but why, emphasizing the global and local impacts of FVW and introducing the concept of waste valorization (composting, donation, processing). Use visual materials and local languages to accommodate low literacy levels. For formal retailers, supermarket corporate head offices should incorporate waste hierarchy principles into ongoing training to challenge the disposal-only mindset.

### 5.2 Interpersonal level

JPC and the COJMM EH Department should establish peer educator programs where trained informal retailers share knowledge with fellow retailers in their markets. Facilitate regular meetings between informal retailer associations and Environmental Health Practitioners to build relationships and trust. Given that 87.3% of informal retailers and 93% of formal retailers indicated their willingness to comply with government officials’ advice regarding FVW management, municipal officials should leverage the high compliance rates observed in both sectors by designating government officials as “trusted messengers” for FVW management guidance, and ensure that such guidance is offered regularly, not just during inspections.

### 5.3 Organizational level

For informal retailers, the COJMM Economic Development Unit should explore collective organizational models, such as retailer cooperatives or market-level associations, that can provide shared resources (e.g., collective waste bins and shared cold storage), training, and waste management services. The COJMM EH Department should develop simple, accessible FVW management guides tailored to informal trading contexts. For formal retailers, retail management should strengthen internal policies to prioritize waste reduction, donation, and valorization over disposal, with clear metrics for monitoring implementation.

### 5.4 Community level

COJMM must urgently provide accessible waste infrastructure (bins, collection points) in areas with high concentrations of informal retailers. Establish local waste drop-off points specifically for FVW, potentially linked to community composting initiatives. Facilitate partnerships between formal retailers and community organizations to donate near-spoiled produce; South African initiatives such as FoodForward SA and Still Good provide replicable models.

### 5.5 Public policy level

Considering that 54.2% of informal retailers reported being unaware of COJMM waste management by-laws, and 54% of formal retailers were uncertain, there is an urgent need to strengthen policy communication. The COJMM Legislative and Policy Unit, in consultation with the national Department of Cooperative Governance, should radically overhaul the communication of COJMM waste management by-laws by using multiple languages, community meetings, local media, and partnerships with retail leaders. The COJMM mayoral committee, with budget allocations from the Municipal Council, must provide tangible support alongside regulations, such as subsidized bins, waste collection services, or training programs, to demonstrate the government’s commitment. The COJMM should consider differentiated approaches that recognize the distinct needs and constraints of informal retailers rather than applying uniform regulations. The National Department of Forestry, Fisheries, and the Environment, in coordination with the COJMM, should accelerate the implementation of South Africa’s Draft Strategy for Reducing Food Losses and Waste with specific targets and support mechanisms for both formal and informal retail sectors. The COJMM Speaker’s office should establish mechanisms for regular consultation between municipal authorities and retailer representatives from both sectors.

## 6 Limitations

This study has several limitations. First, the cross-sectional design captures retailer awareness and practices at a single point in time; therefore, longitudinal research could better track changes following targeted interventions. Second, the quantitative approach, while providing breadth, cannot capture the rich contextual detail that qualitative methods would afford. Third, the study relied on self-reported responses, which may be subject to recall bias and social desirability bias. Fourth, the inferential analysis was limited to univariate chi-square tests of association; future studies with larger samples could employ multivariate techniques to control for confounding variables, and a specialized statistical review would further validate the mathematical robustness of the tests applied. Finally, the study was conducted within a specific municipal region and involved a relatively small sample of formal and informal retailers; therefore, the findings may not be fully generalizable to other municipalities or retail contexts where waste management systems, infrastructure, and regulatory conditions differ.

## 7 Future research

Future studies could employ in-depth interviews or ethnographic observation to understand how informal traders navigate FVW management in their daily practice. Research tracing the actual fate of FVW, where it goes, who handles it, and what happens to it, would complement the focus on retailer perspectives. Intervention research testing the effectiveness of multi-level programmes informed by the SEM would provide evidence for policy and practice. Finally, comparative studies across different South African municipalities could identify contextual factors that influence the effectiveness of various intervention strategies.

## 8 Conclusion

This study has demonstrated that FVW management among retailers in Johannesburg’s Region F is shaped by a complex interplay of factors operating at multiple levels of the social ecology. The stark contrasts between informal and formal retailers, in education, awareness, practices, infrastructure access, and policy engagement, reflect not merely individual differences but systematic disparities in organizational support, community resources, and policy reach. Crucially, both sectors share a fundamental misconception that disposal is the only effective waste management method, revealing a common knowledge gap that transcends sectoral boundaries.

Applying the SEM has enabled a more nuanced understanding than would be possible through a purely descriptive approach. The model reveals that interventions targeting only one level, such as educational campaigns for individuals, are unlikely to succeed when other levels present barriers. Informal retailers face compounded disadvantage across all five levels, while formal retailers, despite their organizational advantages, remain constrained by limited community engagement, policy uncertainty, and the pervasive disposal mindset.

Achieving meaningful reduction in FVW requires moving “away from the bin”, beyond a disposal mentality, toward integrated strategies that address the full spectrum of influences on retailer behavior. These integrated interventions include, but are not limited to, targeted educational campaigns (intrapersonal), peer educator programs (interpersonal), collective infrastructure models for informal retailers and strengthened corporate policies for formal retailers (organizational), provision of waste bins and donation partnerships (community), and improved by-law communication with tangible government support (policy). The findings underscore that addressing FVW in urban African contexts is not merely a technical or educational challenge but a social-ecological one. Effective change requires seeing retailers not as isolated actors but as individuals embedded in complex systems of influence, and designing interventions that engage with the full complexity of their worlds.

## Data Availability

The raw data supporting the conclusions of this article will be made available by the authors, without undue reservation.
